# Charcot arthropathy of the shoulder joint following Arnold-Chiari malformation: A case report

**DOI:** 10.1016/j.ijscr.2025.111741

**Published:** 2025-07-30

**Authors:** Aidin Arabzadeh, Erfan Khosravi, Hamed Naghizadeh, Omid Salkhori, Saleh Validi, Nima Bagheri

**Affiliations:** a Joint Reconstruction Research Center, Department of Orthopedics, Tehran University of Medical Sciences, Iran

**Keywords:** Charcot joint, Neurogenic arthropathy, Arnold-Chiari malformation, Shoulder replacement, Arthroplasty, Syringomyelia

## Abstract

**Introduction:**

Charcot arthropathy of the shoulder is a rare, progressive joint disorder associated with neurosensory deficits, most commonly syringomyelia. It is infrequently linked to Arnold-Chiari malformation, making diagnosis and treatment particularly challenging.

**Case presentation:**

A 51-year-old man presented with chronic, painless swelling and limited range of motion in the right shoulder. He had a history of Arnold-Chiari malformation, treated surgically eight months prior. Imaging revealed extensive humeral head destruction consistent with Charcot shoulder. Reverse shoulder arthroplasty (RSA) was performed due to the advanced stage of joint damage. At one-year follow-up, the patient had an excellent functional recovery, with no postoperative complications or radiographic signs of implant failure.

**Clinical discussion:**

Although rare, Charcot shoulder should be considered in patients with progressive shoulder dysfunction, especially with underlying neurologic conditions. Interestingly, in this case, disease progression continued despite surgical correction of the Arnold-Chiari malformation, suggesting a self-sustaining degenerative process. While RSA is generally used cautiously in Charcot joints due to concerns of instability and bone loss, it proved effective here.

**Conclusion:**

Charcot arthropathy of the shoulder can progress even after resolution of the primary neurologic cause. Early recognition is crucial. In advanced cases, reverse shoulder arthroplasty may offer good functional outcomes when applied in selected patients.

## Highlights

• Charcot shoulder is a rare and underdiagnosed complication of central neurologic disorders such as Arnold-Chiari mal
• This case highlights progression despite surgical correction of the primary neurologic etiology.
• Reverse shoulder arthroplasty (RSA) was selected due to advanced joint destruction and poor rotator cuff integrity.
• RSA led to excellent clinical and radiologic outcomes at one-year follow-up, including near-normal ROM and high patient satisfaction.
• This report supports RSA as a viable option in carefully selected cases of Charcot shoulder.

## 1 Introduction

Neuropathic arthropathy of the shoulder, also known as “Charcot shoulder”, is a very rare, chronic, and progressive disease affecting the shoulder joint [1]. With nearly 60 total cases in the literature, Charcot shoulder is mainly characterized by joint destruction in the presence of a neurosensory deficit [2]. A number of various causes have been noted in the literature, with syringomyelia being considered as the leading cause in the Charcot joints of the upper extremity [3]. Syringomyelia itself can occur as result of malignancy, spinal cord injury, or an Arnold-Chiari malformation [4]. Other conditions such as neurosyphilis, diabetes, chronic alcoholism, and leprosy account for the majority of these cases [3].

Patients with neuropathic arthropathy typically present with painless joint swelling as the primary complaint. Loss of function and joint instability are among other common symptoms. Although imaging in advanced stages of the disease is helpful, the early clinical presentation is usually nonspecific. Both conservative and surgical treatment options have been described in the literature, including nonsteroidal anti-inflammatory drugs (NSAIDs), physical therapy, rehabilitation, arthroplasty, and arthrodesis [5].

However, due to the relatively rare nature of the disease, diagnosis and treatment are still imperfect. In the current study, we aim to present a case of Charcot's shoulder following an Arnold-Chiari malformation, which achieved an ideal prognosis after treatment with reverse shoulder arthroplasty. This article has been written in accordance with the SCARE guidelines [6].

## 2 Case presentation

### 2.1 Patient information

A 51-year-old Middle Eastern male presented to the hospital with complaints of painless swelling and limited range of motion in his right shoulder. The symptoms began 14 months earlier when the patient attempted to lift a heavy object. Initially, he experienced painless swelling in the shoulder, without any associated systemic symptoms such as fever. The patient was a worker, right-handed, and had a body mass index of 26.2.

The patient's past medical history included hypertension and Arnold-Chiari malformation, for which he underwent surgical treatment 8 months prior. His surgical history also included an appendectomy and herniorrhaphy. At the time of presentation, he was taking Losartan 25 mg twice daily, with no reported allergies or relevant habitual history.

### 2.2 Clinical findings

On clinical examination, the patient was conscious, oriented, and had stable vital signs. The neurovascular examination was unremarkable. Asymmetry of the shoulders was observed, with noticeable atrophy of the muscles in the right shoulder. Palpation revealed an empty glenoid fossa.

**Fig. 1:**
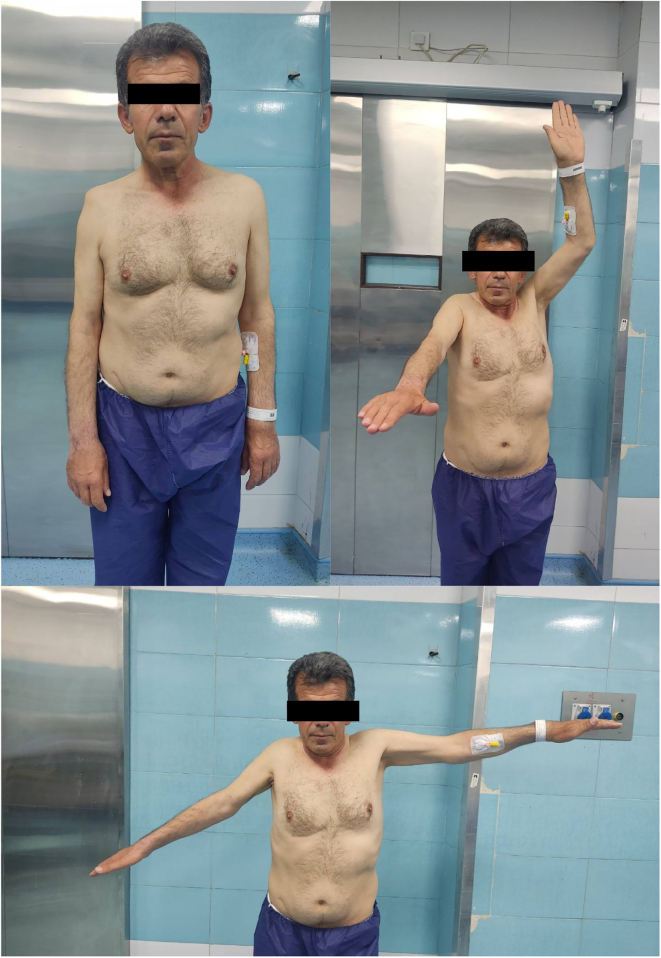
Clinical examination of the patient shows asymmetry in active movements and muscle atrophy in the right shoulder, along with limited abduction and forward flexion.

**Fig. 2:**
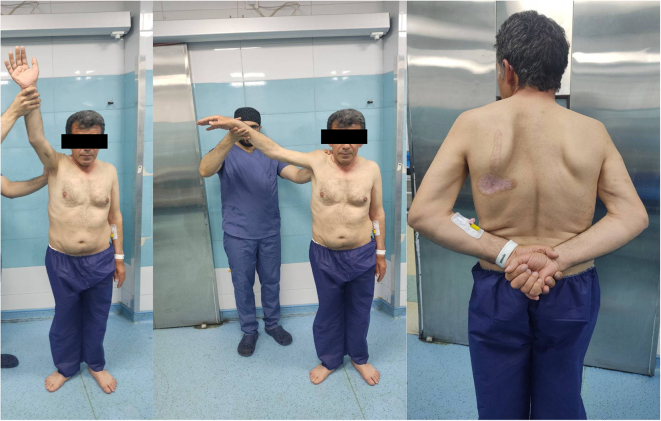
Clinical examination of the patient showing preserved range of motion in internal rotation and passive movements of the right shoulder.

Active forward elevation and abduction were both limited to 70 degrees, while passive forward elevation and abduction were intact at 180 degrees. Internal rotation and external rotation were preserved without limitation (Fig. 1, Fig. 2). No pain or tenderness was noted in the right shoulder, and there were no clinical signs suggestive of septic arthritis. Neurological examination revealed symmetrical deep tendon reflexes with no evidence of asymmetry.

### 2.3 Diagnostic workup

The patient mentioned that he had undergone a shoulder radiograph at another center one year prior (2 months after symptom onset), which reportedly showed humeral destruction; however, the image was unavailable for review. No further diagnostic evaluation had been performed at that time.

**Fig. 3:**
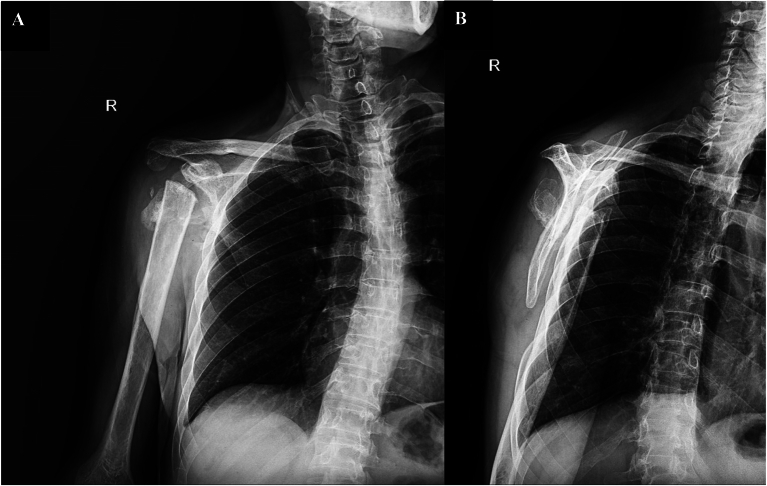
Radiographic image (anteroposterior (A) and scapular Y-view (B)) of the right shoulder showing extensive bone resorption in the right humeral head and joint destruction consistent with Charcot shoulder.

**Fig. 4:**
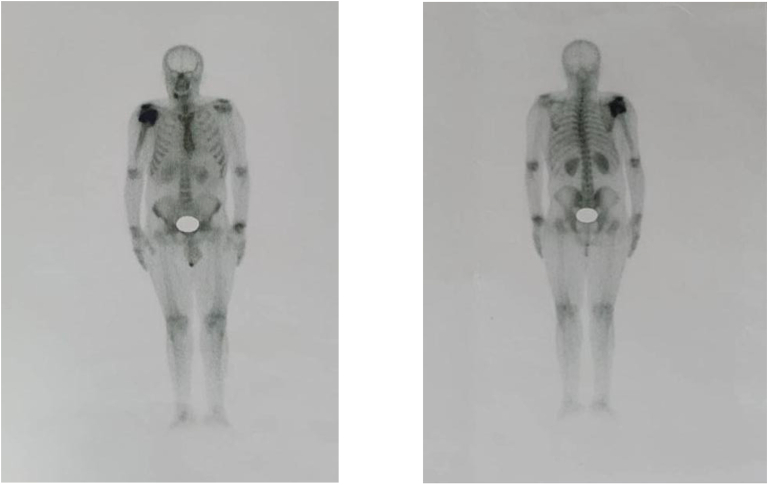
Whole-body bone scan demonstrating intense increased tracer uptake in the right humeral head and diffuse increased tracer uptake in the proximal and mid shaft of the right humerus, indicative of active joint destruction.

At our center, anteroposterior (AP) and scapular Y-view radiographs showed extensive bone resorption in the right humeral head and significant joint destruction (Fig. 3). In addition, a whole-body bone scan showed intense increased tracer uptake in the right humeral head and diffuse increased tracer uptake in the proximal and midshaft of the right humerus (Fig. 4).

### 2.4 Therapeutic intervention

In our case, reverse shoulder arthroplasty (RSA) was selected over other potential treatment options such as arthrodesis or conservative management. This decision was based on the patient's relatively high level of activity and functional demands, along with her expressed desire for restored mobility. Additionally, due to severe proximal humeral bone resorption and the inability to reconstruct the rotator cuff (because of the complete resorption of the tuberosities), RSA offered the best chance for functional restoration in the setting of rotator cuff insufficiency and preserved deltoid function, as supported by recent literature identifying RSA as the preferred option for advanced Charcot arthropathy of the shoulder with structural compromise.

The patient underwent reverse shoulder arthroplasty using an Aequalis Reversed Fracture Shoulder Prosthesis (Tornier/Stryker) with a 13-centimeter stem. The implant features a low-profile metaphyseal component with hydroxyapatite coating, a grafting window, and modular polyethylene and glenoid options. The surgical procedure was performed by a fellowship-trained shoulder surgeon with over 10 years of experience in shoulder arthroplasty.

**Fig. 5:**
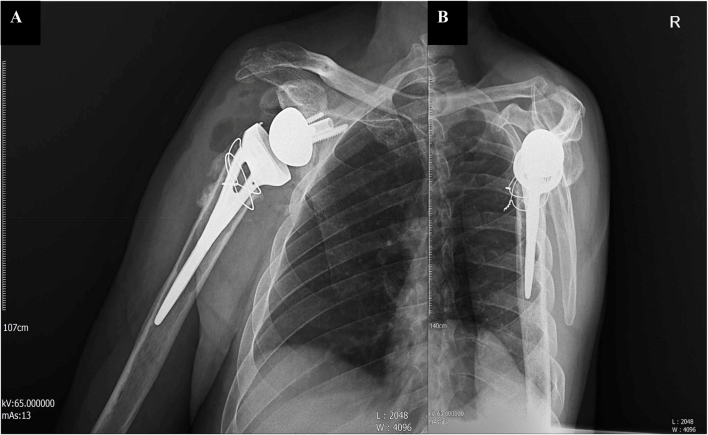
Postoperative anteroposterior (A) and Y-view (B) radiographs showing the reverse shoulder arthroplasty (RSA) in place.

The deltopectoral approach was carried out, with stem placement in 30 degrees of retroversion. A guide pin was placed at 10–15 degrees of inferior tilt. After confirming the adequate inferior tilt of the baseplate, it was impacted and secured with screws. The Morse taper was dried, and the glenosphere was impacted into position. After dislocating the glenohumeral joint, the humeral stem was placed, and the joint was reduced. Next, for delivering the bearing surface, the joint was re-dislocated and reduced once more. Finally, the wound was closed after subscapularis repair (Fig. 5) [7].

### 2.5 Follow-up and outcomes

**Fig. 6:**
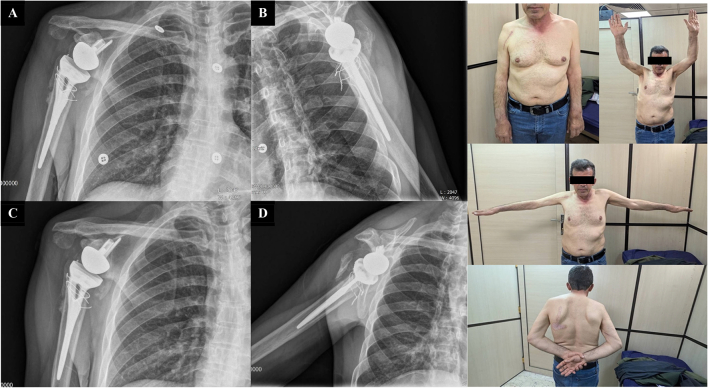
Serial follow-up radiographs following the reverse shoulder arthroplasty, at 3-month (A) and (B) and 6-month (C) and (D) follow-up, with no signs of prosthetic dislocation, loosening, or acromial/periprosthetic fractures. At 6-month follow-up Clinical photographs demonstrate that the patient has regained near-complete range of motion.

**Fig. 7:**
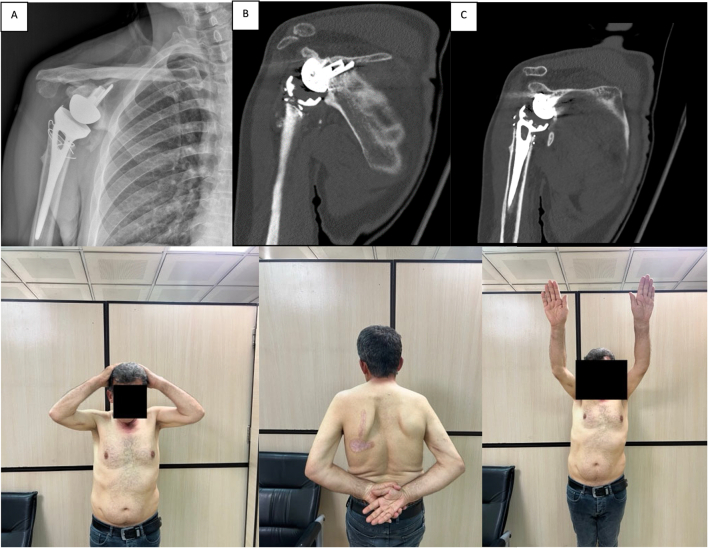
At one-year follow-up, radiographic and CT evaluations revealed no evidence of dislocation, component loosening, or any other abnormalities. Clinical photographs demonstrate that the patient has regained near-complete range of motion.

The patient demonstrated an excellent prognosis with a full return of joint range of motion. Postoperative and serial follow-up radiographs, taken over a period of six months and one year, showed no evidence of prosthetic dislocation, loosening, acromial fractures, or periprosthetic fractures (Fig. 6, Fig. 7). The patient's one-year follow-up CT scan showed no evidence of prosthesis loosening. The range of motion was preserved at the 6-month and one-year follow-up (Fig. 6, Fig. 7). Functional evaluation using the DASH (Disabilities of the Arm, Shoulder and Hand) questionnaire revealed a score of 8.3, indicating a satisfactory outcome.

The patient reported high satisfaction with the surgical outcome. Her self-reported satisfaction score on the Visual Analog Scale was 90 out of 100, reflecting a very favorable perception of the treatment and its results. Table 1 presents a timeline of key clinical events, outlining the patient's progression from symptom onset to one-year postoperative follow-up (Table 1).

**Table 1:** Chronological summary of key clinical events from symptom onset to final follow-up. This timeline illustrates the patient's clinical course, including initial presentation, surgical intervention, and follow-up evaluations demonstrating functional recovery and implant stability.

Timepoint	Clinical event
Month −14	Onset of shoulder pain and swelling after lifting a heavy object
Month −8	Surgical treatment for Arnold-Chiari malformation
Month 0	Presentation to our center; imaging and diagnosis of Charcot shoulder
Week 2 (post-presentation)	Reverse shoulder arthroplasty performed
Month 3	Follow-up radiographs showed no complications
Month 6	Follow-up radiographs and clinical exam: ROM improved
Month 12	X ray and CT scan confirmed no loosening; near-full ROM; DASH score 8.3

## 3 Discussion

Although previous reports have described the association between Charcot arthropathy and Arnold-Chiari malformation, this case is unique in demonstrating that significant shoulder joint destruction can still occur and progress despite prior surgical treatment of the underlying neurological condition. Furthermore, to our knowledge, few cases have documented the use of reverse shoulder arthroplasty in this context, with favorable functional outcomes, including near-complete range of motion, a low DASH score (8.3), and a high patient satisfaction score (VAS 90/100). This report highlights the importance of long-term musculoskeletal surveillance and reinforces the potential role of RSA in selected neuroarthropathic cases.

Diagnosing Charcot arthropathy of the shoulder presents considerable challenges due to its rarity, atypical presentation, and often painless course despite significant joint destruction. Early radiographic findings may be subtle or non-specific, and the lack of pain, a hallmark of neuropathic joints, can mislead clinicians to overlook serious pathology. In many cases, patients present with minimal discomfort, joint swelling, and limited range of motion, which can be mistaken for more common inflammatory or degenerative conditions [[8], [9], [10]].

Additionally, in patients with underlying neurological disorders such as syringomyelia or Arnold-Chiari malformation, musculoskeletal symptoms may precede overt neurological signs, further complicating timely diagnosis. Advanced imaging, particularly MRI, plays a crucial role in identifying associated spinal cord pathology, while CT and plain radiographs help assess joint destruction [1,[11], [12], [13], [14]].

The differential diagnosis of Charcot shoulder includes a wide range of conditions that can lead to rapid joint destruction and shoulder dysfunction. These include septic arthritis, neoplastic lesions (such as primary bone tumors or metastases), advanced osteoarthritis, inflammatory arthropathies (like rheumatoid arthritis), and crystal-induced arthropathies such as gout or calcium pyrophosphate deposition disease [1,2,10,[14], [15], [16]].

In the context of neurologically compromised patients, it is especially important to rule out infection and neoplasm through laboratory tests, joint aspiration (if indicated), and appropriate imaging. Furthermore, in syringomyelia-associated cases, the lack of systemic signs of infection and characteristic findings on spinal MRI can help distinguish Charcot arthropathy from other destructive arthropathies. Early consideration of neuropathic joint as a potential diagnosis is essential to prevent further deterioration and initiate appropriate management [[8], [9], [10], [11], [12], [13],^17^,^18^].

With a mean age at diagnosis of nearly 50 years, our patient had a relatively typical presentation with initial symptoms of pain and swelling, which progressed into a painless limitation in ROM. Approximately 27 % of Charcot shoulder cases have been followed by shoulder trauma [5]. Patients with a history of trauma generally have a shorter time to presentation, with an average of 519 days, which aligns with the timeline observed in this case [5]. Although our patient did not experience overt trauma as per the orthopedic definition, excessive joint loading may have included microtrauma on the structures of the joint, contributing to disease progression. The patient's initial symptoms of pain and swelling support this hypothesis.

Interestingly, the disease progression continued despite the surgical correction of the Arnold-Chiari malformation. Literature suggests that Arnold-Chiari malformation was likely the primary etiologic factor leading to the development of the Charcot joint in this case [19]. However, the persistence of disease progression after treatment of the underlying etiology raises the possibility that the initiation of the disease depends on the primary etiology, while its progression may occur independently, forming a self-perpetuating vicious cycle. Whether conservative interventions can interrupt this cycle and prevent further progression following the resolution of the primary etiology remains an open question requiring further research [19].

This phenomenon is thought to result from persistent or irreversible damage to central sensory pathways, particularly the spinothalamic and dorsal columns, which impairs protective pain and proprioceptive feedback. As a result, repetitive microtrauma to the joint can go unnoticed, leading to progressive degeneration. Additionally, neurovascular mechanisms involving sympathetic dysfunction may further exacerbate bone resorption and joint instability. Therefore, the development of Charcot arthropathy in such cases reflects the complex and often irreversible neurophysiological alterations that can persist even after the primary neurologic pathology has been addressed [1,2,10,13,14,16].

The relatively late presentation of our case, with advanced disease progression and complete joint destruction, highlights the need for increased awareness of Charcot shoulder in clinical practice. It is essential to include Charcot shoulder in the differential diagnosis for patients presenting with chronic, progressive shoulder symptoms, as early stages of the disease are often underdiagnosed [20]. Additionally, some cases may present with atypical histories of trauma, such as heavy loading. It is important to consider the potential role of microscopic trauma in the initiation or progression of disease.

The literature points to various treatment options, ranging from conservative management to surgical intervention [5,13,16,21]. In our case, since the disease had led to the destruction of the shoulder, conservative treatment strategies were no longer an option. RSA is generally contraindicated in Charcot shoulder due to concerns over mechanical instability, progressive joint destruction, and significant bone loss. However, recent studies suggest that the relative indications for RSA in challenging cases like Charcot shoulder are evolving, with emerging evidence supporting its potential efficacy under carefully selected circumstances [22].

Performing RSA in patients with Charcot neuroarthropathy presents specific challenges, primarily due to poor bone quality, loss of proprioception, and an increased risk of implant-related complications. Studies have reported higher incidences of glenoid loosening, instability, and periprosthetic fractures in this population. Intraoperatively, meticulous assessment of glenoid bone stock is crucial, and augmented baseplates or longer stems may be necessary in cases of severe bone loss. Additionally, postoperative management must consider the lack of protective sensation, which predisposes the joint to microtrauma and unrecognized stress. As highlighted by Park et al. [2], even with well-positioned implants, early complications such as glenoid fractures and dislocation can occur, necessitating staged revisions. These considerations underscore the need for close, long-term follow-up and patient education regarding joint protection [2,14,15,22].

In this case, RSA demonstrated remarkable success in restoring shoulder function, even in the presence of these challenges. Our patient achieved a favorable outcome without radiological evidence of complications, such as glenoid loosening, periprosthetic fractures, or acromial stress fractures, which are often cited as potential complications in this population.

## 4 Conclusions

As a rare destructive pathology, Charcot shoulder can occur in individuals with a variety of underlying conditions, spanning from spinal abnormalities to diabetes. It is important to note that disease progression may persist even after addressing the underlying etiology, emphasizing the importance of including Charcot shoulder in the differential diagnosis for patients with atypical clinical presentations and nonspecific radiographic findings. Treatment strategies for Charcot shoulder are still evolving. Still, treatment should be personalized according to the degree of functional limitation and underlying etiology.

## CRediT authorship contribution statement

Nima Bagheri, M.D.: Conceived and designed the study, led the research team and contributed to manuscript revision.
Aidin Arabzadeh, M.D.: Assisted in study design, collected the primary data, and contributed to data analysis.
Hamed Naghizadeh, M.D.: Conducted data analysis, interpreted the data and drafted the manuscript.
Omid Salkhori, M.D.: Provided critical revisions that were important for the intellectual content, and final approval of the version to be published.
Saleh Validi, M.D.: Assisted in data collection and analysis, and contributed to drafting and revising the manuscript.
Erfan Khosravi, M.D.: Coordinated the research efforts, ensured the integrity of the work, and contributed to manuscript preparation and submission.

## Informed consent

The patient provided informed written consent for print and electronic publication of this case report.

## Ethical approval

Ethical approval is not required.

## Guarantor

Nima Bagheri M.D. agrees to accept full responsibility for the work and the conduct of the study, has access to the data, and controls the decision to publish.

## Research registration number

This study does not qualify as a “First in Man” study and therefore did not require registration.

## Research location

Orthopedic Surgery Department, Imam Khomeini Complex Hospital, Tehran University of Medical Sciences, Tehran, Iran.

## Funding

There was no funding source for this study.

## Declaration of competing interest

All authors listed above mentioned that there is no conflict of interest in this study, and no benefits in any form have been or will be received from a commercial party related directly or indirectly to the subject of this manuscript.
